# A Bayesian Failure Prediction Network Based on Text Sequence Mining and Clustering

**DOI:** 10.3390/e20120923

**Published:** 2018-12-03

**Authors:** Wenbing Chang, Zhenzhong Xu, Meng You, Shenghan Zhou, Yiyong Xiao, Yang Cheng

**Affiliations:** 1 School of Reliability and System Engineering, Beihang University, Beijing 100191, China; 2 Center for Industrial Production, Aalborg University, 9220 Aalborg, Denmark

**Keywords:** textual data, word2vec, CFSFDP, PrefixSpan, Bayesian failure network

## Abstract

The purpose of this paper is to predict failures based on textual sequence data. The current failure prediction is mainly based on structured data. However, there are many unstructured data in aircraft maintenance. The failure mentioned here refers to failure types, such as transmitter failure and signal failure, which are classified by the clustering algorithm based on the failure text. For the failure text, this paper uses the natural language processing technology. Firstly, segmentation and the removal of stop words for Chinese failure text data is performed. The study applies the word2vec moving distance model to obtain the failure occurrence sequence for failure texts collected in a fixed period of time. According to the distance, a clustering algorithm is used to obtain a typical number of fault types. Secondly, the failure occurrence sequence is mined using sequence mining algorithms, such as-PrefixSpan. Finally, the above failure sequence is used to train the Bayesian failure network model. The final experimental results show that the Bayesian failure network has higher accuracy for failure prediction.

## 1. Introduction

As a large-scale complex equipment system, an aircraft is composed of flight control systems, avionics systems, and power systems. The long service life of aircraft and the complicated and harsh flight environment have resulted in frequent aircraft failures. Unstructured text data is very common in practical situations, and such data is often ignored by data users. A large amount of text data recorded in natural language has been accumulated during aircraft maintenance. The data accumulated over the years is easy to acquire and does not require complex specialized equipment for acquisition. If these text data can be adequately utilized, it will greatly promote the maintenance and protection process. Traditional failure prediction is mainly based on structured data. Failure prediction and diagnosis based on text data is a novel field. The failure mentioned here refers to failure types, such as transmitter failure and signal failure, which are classified by the clustering algorithm based on the failure text.

In Choi’s 2018 paper [1], failure load prediction for composite joints with clamping force was conducted using a characteristic length method combined with Tsai-Wu failure criteria. Valis et al. [2]. focused on the system of piston combustion engine and tribodiagnostic data for soft and hard failure prediction. The data also includes numerical data, which is structural. Moreover, Abu-Samah et al. [3]. presented a methodology to extract and validate rules (and patterns) as time bound failure signatures. According to the failure signatures, then it used the Bayesian approach was then to predict real time failure. In comparison to existing approaches to learn and extract failure signatures, the presented methodology offers the extraction, selection and validation of rules/patterns. This methodology is employed to execute corrective and proactive measures to avoid failures within a certain period of time. In the medical field, fault prediction based on structured data is already mature. Mdhaffar et al. [4]. presented a novel health analysis approach for heart failure prediction. It is based on the use of complex event processing (CEP) technology, combined with statistical approaches. The prediction model works well. For failure prediction, forecasting technology based on structured data is already mature. However, there is not much research related to making predictions based on text data. Lee’s 2015 research [5] mainly dealt with the analysis of films’ box office success or failure using text mining. For data, it used a portal site and film review data, grade point average and the number of screens gained from the Korean Film Commission. The purpose of their paper was to propose a model to predict whether a film will be successful or not using these aforementioned data. 

For failures, research using textual data for prediction is currently rare. In this article, the data is fault text data with a time-series relationship. The occurrence of these failures has a temporal relationship. When a failure occurs, it may cause another failure. This paper uses a series of algorithms to build a fault prediction model to achieve fault prediction based on this data.

In fact, Chinese texts are different from English texts. Words are separated by spaces in English texts. First, word segmentation and stop words are first applied to the original textual failures data. Word2vec uses text vectorization to calculate cosine similarity. Then, clustering algorithms classify failures into several classes based on data characteristics. Finally, the Bayesian network is used to build the dependencies between the above mentioned several types of faults and to predict failure. The innovation of this paper is the proposition of a failure prediction method based on textual data. This approach will greatly promote the maintenance and protection process. The relationship between some failures can be found at the data level. However, such relations are difficult to identify at the mechanism level. Finally, this approach can also provide guidance for exploring the cause of the failure mechanism.

## 2. Data Processing

The unstructured data present in aircraft maintenance must first be preprocessed. Different from the structured data processing method, this paper uses the knowledge of natural language processing to process text data. In English texts, words are separated by spaces, but for Chinese texts, there are no obvious separators in the text. Before the text representation process, word segmentation needs to be completed. The common Python Chinese word segmentation system mainly includes the jieba Chinese word segmentation system, Chinese Academy of Sciences word segmentation system, smallseg, and snailseg. A functional support comparison of these systems is shown in Table 1.

This paper adopts jieba to complete segmentation work. In addition, the Chinese texts need to stop words like “,” and “ah”. This paper adopt the unstructured data in time series. Data preprocessing results are shown in Table 2.

## 3. Methodology

### 3.1. Word2vec Moving Distance Model

There are two main methods used for text vectorization: word2vec and doc2vec. Word2vec only performs “semantic analysis” based on the dimension of the word, and does not have the contextual capability of “semantic analysis”. The text data in this paper is mainly the name of the fault, not the description of the fault with the context.

Reference [6] adopted word2vec. Word2vec was also employed in 2013 as an efficient tool for Google to express words as real-valued vectors. Kai et al. [7]. argued that the domain knowledge is reflected by the semantic meanings behind keywords rather than the keywords themselves. They applied the word2vec model to represent the semantic meaning of the keywords. Based on that work, they proposed a new domain knowledge approach, the semantic frequency semantic active index, similar to the frequency-inverse document frequency, to link domain and background information and to identify infrequent but important keywords. Park et al. [8]. suggested an efficient classification method of Korean sentiment using word2vec and recently studied ensemble methods. For the 200,000 Korean movie review texts, they generated a POS (Part Of Speech)-based BOW (Bag Of Words) feature and a feature using word2vec, and integrated all the features of the two feature representations.

Yongjun et al. [9]. examined the ability of word2vec to derive semantic relatedness and similarity between biomedical terms from large publication data. They downloaded abstracts of 18,777,129 articles from PubMed and 766,326 full-text articles from PubMed Central (PMC). The datasets were preprocessed and grouped into subsets by recency, size, and section. Word2vec models were trained on these subtests. Cosine similarities between biomedical terms obtained from the word2vec models were compared against reference standards. The performance of models trained on different subsets were compared to examine recency, size, and section effects. To extract key topics from new articles, Zhao et al. [10]. researched into a new method to discover an efficient way to construct text vectors and improve the efficiency and accuracy of document clustering based on the word2vec model. Through training, the processing of text content was reduced to *K*-dimensional vector operations, and the similarity in vector space can be used to represent the semantic similarity of text. The word2vec word vector model includes the CBOW (Continuous Bag-of-Words Model) model and Skip-gram model. It can be designed based on Hierarchical Softmax and Negative Sampling algorithms.

**Figure 1:**
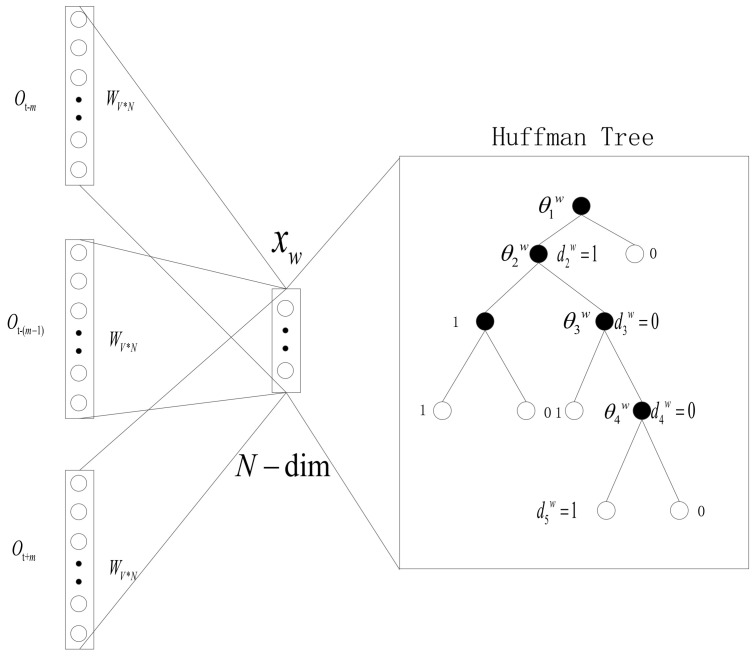
Hierarchical Softmax CBOW Model Schematic.

The schematic diagram of the CBOW model based on the Hierarchical Softmax is shown in Figure 1. It is composed of three layers: input layer, projection layer, and output layer. Here, we take the sample (Context(w),w) (with m words before and after *w*) as an example to explain.

Input layer: One-hot representation with a total of 2 m words in context. There are 2m×V nodes $o_{t-m},o_{t-\left(m-1\right)},\cdots ,o_{t-1},o_{t+1},\cdots o_{t+\left(m-1\right)},o_{t+m}\in R^{V}$.

Projection layer: Accumulating sum of 2 m vectors in the input layer: $x_{w}=\sum_{i=1}^{2m}v\left(Context{\left(w\right)}_{i}\right)\in R^{N}$ with a total of *N* nodes.

Output layer: A Huffman tree using the word frequency of each word in the corpus as its weight. Its leaf nodes are all words appearing in the corpus. There are a total of *V* leaf nodes, corresponding to each word in dictionary D, and there are *V* − 1 non-leaf nodes.

Among them, there is a word matrix $W_{V\times N}$ from the input layer to the projection layer. The matrix is essentially the output form of the word vector after training.

The word vector matrix $X\in {\mathbb{R}}^{d\times N}$ can be obtained by word2vec, where *N* represents the dictionary consisting of *N* words, and d represents the dimension of the word vector. The *i*-th column in the matrix, the column vector $x_{i}\in R^{d}$, represents the word vector of the *i*-th word $w_{i}$ in the dictionary in the *d*-dimensional space.

The idea of the word vector moving distance model is that each word vector in the text can be partially or completely transformed into a word vector in the text, that is, each word in one text is matched to all words in the other text with different weights.

*Standardized word bag representation*: $d_{w_{i}}=\frac{tf\left(w_{i},S_{1}\right)}{\sum_{i=1}^{m}tf\left(w_{i},S_{1}\right)}$. $tf\left(w_{i},S_{1}\right)$ represents the frequency of word $w_{i}$ in text $S_{1}$ which has *m* different words.

*Word vector moving cost*: The goal is to combine the degree of semantic similarity between word pairs into the text distance matrix. The Euclidean distance $c\left(w_{i},w_{j}\right)={\left\|x_{i}-x_{j}\right\|}_{2}$ between $w_{i}$ and $w_{j}$ is the word vector moving cost.

*Word vector moving distance*: $T\in R^{m\times n}$ is a flow matrix and $T_{w_{i}w_{j}}\geq 0$ represents the number of the *i*-th word in text $S_{1}$, which flows to the *j*-th word in text $S_{2}$. For the purpose of fully transforming text $S_{1}$ into text $S_{2}$, it should be guaranteed that the sum of the outflow *i*-th word should be equal to $d_{w_{i}}$, $\sum_{j}T_{w_{i}w_{j}}=d_{w_{i}}$, and the inflow of the *j*-th word should be equal to $d_{w_{j}}$, $\sum_{i}T_{w_{i}w_{j}}=d_{w_{j}}$. The distance between text $S_{1}$ and text $S_{2}$ can be represented by the minimum cumulative cost of the word moving from text $S_{1}$ to $S_{2}$.

A word vector movement distance model is created as follows:

(1) $$\underset{T\geq 0}{\min}\sum_{i=1,j=1}^{m,n}T_{w_{i}w_{j}}c\left(w_{i},w_{j}\right)$$

Subject to:

(2) $$\sum_{j=1}^{n}T_{w_{i}w_{j}}=d_{w_{i}}\forall i\in \left\{1,\cdots ,m\right\}$$

(3) $$\sum_{i=1}^{m}T_{w_{i}w_{j}}=d_{w_{j}}\forall j\in \left\{1,\cdots ,n\right\}$$

(4) $$d_{w_{i}}=\frac{tf\left(w_{i},S_{1}\right)}{\sum_{i=1}^{m}tf\left(w_{i},S_{1}\right)}$$

(5) $$d_{w_{j}}=\frac{tf\left(w_{j},S_{2}\right)}{\sum_{j=1}^{n}tf\left(w_{j},S_{2}\right)}$$

The algorithm complexity is $O\left(p^{3}\log p\right)$, where *p* represents the number of different words.

Based on the distance of the word vector of the two texts, the text similarity of the two texts can be calculated by normalizing the moving distance of the word vector between the texts. This is calculated as follows:

(6) $$similarity\left(S_{1},S_{2}\right)=1-\frac{WMD\left(S_{1},S_{2}\right)-\min\left(WMD\right)}{\max\left(WMD\right)-\min\left(WMD\right)}$$

min(WMD) and max(WMD), respectively, represent the minimum word vector movement distance and the maximum word vector movement distance in the data set.

Because of the similarity measure characteristics, the similarity matrix is a symmetric matrix with 1 on the diagonal, where the range of elements is (0,1). If the similarity of two texts is greater, then the distance will be smaller. On the contrary, the smaller the similarity, the greater the distance. Therefore, the final distance between two texts is the reciprocal of their similarity.

### 3.2. Clustering Algorithm for Failure Type

The *k*-means method [11] is a classical method used to solve the clustering problem. The algorithm is very subjective and requires the number of clusters which are specified in advance. Many clustering algorithms have been developed, such as grid-based [12], hierarchy-based [13], model-based [14], and density-based [15] clustering algorithms. The processing time of the grid clustering algorithm is related to the number of cells divided by each dimensional space, which reduces the quality and accuracy of clustering. The computational complexity of the hierarchy-based algorithm is too high. The model clustering algorithm is based on the hypothesis: that variables are independent of each other. However, this assumption is often not true. For the density-based clustering algorithm, when the density distribution is not uniform, the clustering effect is worse. The clustering algorithm used in this paper is a new clustering algorithm proposed by Rodriguez and Laio [16] in “*Science*”, which is novel and simple and fast. According to the characteristics of the data, the clustering algorithm can automatically determine the number of cluster centers. The clustering effect and computational efficiency are very high. There are two basic assumptions in the clustering algorithm:

1. There are points with a lower density than the clustering center
2. These points are less distant from the cluster center than other cluster centers.

This clustering algorithm can be divided into four steps. Here is a brief introduction to these four steps:

1. Calculate the local density

The clustering set is $S={\left\{x_{i}\right\}}_{i=1}^{N}$. This paper adopts the Gaussian kernel function to calculate the density. The formula is as follows:

(7) $$\rho_{i}=\underset{j\epsilon I_{s\backslash \left[i\right]}}{\sum }e^{-{\left(\frac{d_{ij}}{d_{c}}\right)}^{2}}$$

where $\rho_{i}$ is the number of data points whose distance is less than *d_c_*, regardless of the value of *x_i_* itself. $I_{s}=\left\{1,2,\ldots ,N\right\}$ is an indicator set. $d_{ij}=\;dist\left(x_{i},x_{j}\right)$ represents the distance between points $x_{i}$ and $x_{j}$. The parameter d_c_ should be specified in advance. To some extent, this parameter *d_c_* determines the effect of the clustering algorithm. If *d_c_* is too large, the local density value of each data point will be large, resulting in low discrimination. The extreme case is that the value of *d_c_* is greater than the maximum distance of all points, so the end result of the algorithm is that all points belong to the same cluster center. If the value of *d_c_* is too small, the same group may be split into multiples. The extreme case is that *d_c_* has a smaller distance than all points, which will result in each point being a cluster center. The reference method given by the author in this paper is to select a *d_c_* so that the average number of neighbors per data point is about 1–2% of the total number of data points.

2. Calculate the distance

A descending sequence of subscripts ${\left\{q_{i}\right\}}_{i=1}^{N}$ is generated:

(8) $$\rho_{q1}\geq \rho_{q2}\geq \cdots \geq \rho_{qN}$$

The distance formula is as follows:

(9) $$\delta_{i}=\{\begin{array}{c} \underset{\begin{array}{c} qj\\ j<i \end{array}}{\min}\left\{d_{qiqj}\right\},\;i\geq 2;\\ \underset{j\geq 2}{\max}\left\{\delta_{qj}\right\},\;i=1. \end{array}$$

For the above formula, when *i* = 1, $\delta_{i}$ is the distance between the data point with the largest distance from *x_i_* in *S*. If i≥2, $\delta_{i}$ represents the distance between *x_i_* and the data point (or those points) with the smallest distance from *x_i_* for all data points with a local density greater than *x_i._*

3. Select the clustering center

So far, the ($\rho_{i},\delta_{i}),i\in I_{s}$ of every data point can be achieved. For the comprehension consideration, we use the following formula to select the clustering center:

(10) $$\gamma_{i}=\rho_{i}\delta_{i},i\epsilon I_{s}$$

**Figure 2:**
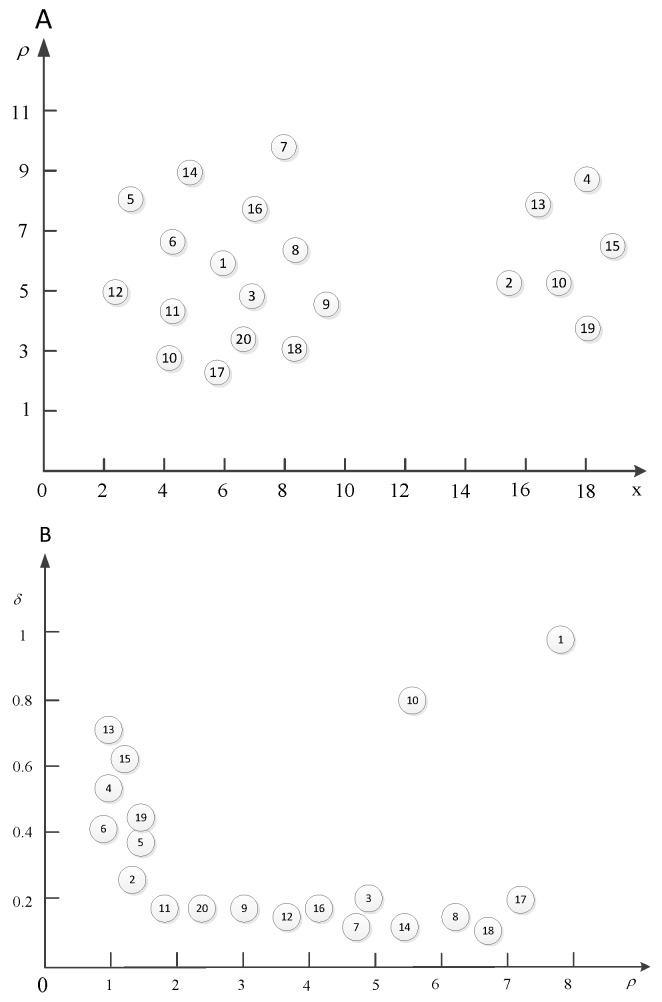
Example and schematic. (**A**) Clustering effect diagram; (**B**) Cluster centers selection.

For example, the following figure (Figure 2) contains 20 data points. We already have got the ($\rho_{i},\delta_{i}),i\in I_{s}$ of every data point.

As shown in Figure 2, Figure (A) is the clustering effect diagram of data points. Data points are divided into two clusters. The center of first cluster is data point 1 while the center of the second cluster is data point 10. These two clustering centers are selected according to Figure (B). In Figure (B), ρ is the number of data points whose distance is less than this point. δ is the distance between the data points. From Figure (B), we can see that data points 1 and 10 are far away from other points in the coordinate system. According to the core idea of this clustering algorithm, clustering centers are those with many data points around them and are far away from other clustering centers. Therefore, data points 1 and 10 are the clustering centers in this case.

**Figure 3:**
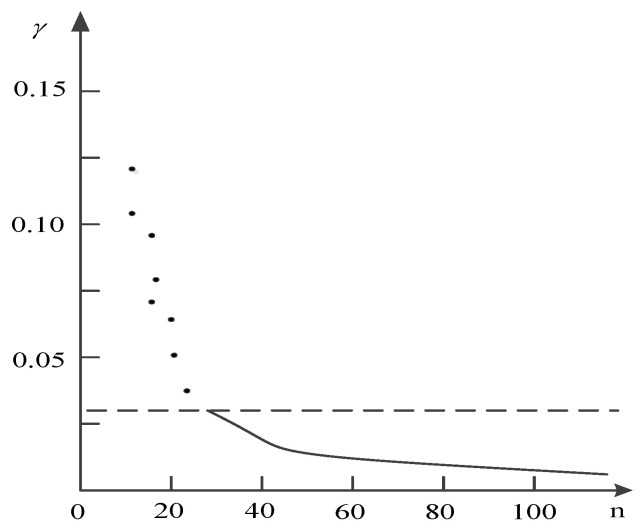
γ curve.

Next, we calculate the γ to select the cluster center. The following figure (Figure 3) is the γ curve.

According to this figure, it was found that the curve is smoother for the non-cluster centers. Furthermore, there is a clear jump between the cluster centers and non-cluster centers.

4. Categorize other data points

According to the cluster center, the distance between the cluster center and the data points can be calculated. The data points are classified into the cluster center which is closest to each data point.

### 3.3. Failure Sequence Mining Algorithm—PrefixSpan

Common sequence pattern algorithms are the Generalized Sequential Pattern (GSP mining algorithm), Apriori, CloSpan and PrefixSpan. GSP and Apriori are the traditional algorithms for sequence mining and their performances are worse than that of PrefixSpan. CloSpan is suitable for long serial text mining. In terms of short sequence mining, PrefixSpan is better.

This paper’s text data sequence is shorter, so this paper adopts the PrefixSpan algorithm [17]. PrefixSpan is a kind of sequential pattern mining algorithm. PrefixSpan has been applied in many fields. For example, it is applied in the mining process for the Indonesian language, which continues to be an interesting research topic. Maylawati et al. [18]. compared several sequential pattern algorithms, including BI-Directional Extention (BIDE), PrefixSpan, and TRuleGrowth. They founded that the average processing time of PrefixSpan was faster than those of BIDE and TRuleGrowth. On the other hand, PrefixSpan and TRuleGrowth were more efficient in using memory than BIDE.

In order to solve the problem of large space and time overhead in the PrefixSpan algorithm, a new sequential pattern mining algorithm based on PrefixSpan is proposed, termed as the PrefixSpan-*x* algorithm. This algorithm [19] reduces unnecessary storage space and removes the non-frequent items. PrefixSpan has also been applied to big data. To support PrefixSpan scalability, there exist two problems regarding its implementation in a MapReduce framework. The first problem is related to parsing and analyzing big data, while the second is related to managing projected databases. In this paper, we propose two methods, i.e., Multiple MapReduce and Derivative Projected Database to overcome the first and the second problems. Sambrina et al. [20]. Showed that those proposed methods can significantly reduce execution time in supporting the scalability of PrefixSpan.

A sequence database *S* is a collection of different sequences, while s is a sequence of it. The sequence $\alpha =\{a_{1}a_{2}\ldots a_{n}\}$ is the subsequence of $s=\{b_{1}b_{2}\ldots b_{m}\}$ which also indicates that sequence s includes α, α⊆s. If there is an integer $1\leq j_{1}<j_{2}<\ldots <j_{n}<m$, make $a_{1}\subseteq b_{j_{1}}$, $a_{2}\subseteq b_{j_{2}}$, …, $a_{n}\subseteq b_{j_{n}}$. The degree of support for the sequence α in the sequence database *S* is the number of sequences containing the sequence α in the sequence database *S*, denoted as Support(α). Given the support threshold min_sup, if the support of the sequence α in the sequence database is not less than min_sup, the sequence α is called sequence mode. Among them, a sequence pattern with length l is denoted as l-mode.

**Definition** **1.** *Prefix: Set all items in each element of the sequence in lexicographic order. The sequences* $\alpha =<e_{1}e_{2}\ldots e_{n}>$ *,* $\beta =<e_{1}{}^{\prime }e_{2}{}^{\prime }\ldots e_{n}{}^{\prime }>$ *(m < n) are given. If* $e_{i}{}^{\prime }=e_{i}(i\leq m-1)$ *,* $e_{m}{}^{\prime }\subseteq e_{m}$ *and* $\left(e_{m}-e_{m}{}^{\prime }\right)$ *items are behind the project in* $e_{m}{}^{\prime }$ *, then* β *is the prefix of* α *.*

**Definition** **2.** *Projection: Given the sequence* α *and* β *, if* β *is the subsequence of* α *,* $\alpha^{’}$ *which was the projection of* β *for* α *must meet the following constraints:* β *is the projection of* $\alpha^{’}$ *and* $\alpha^{’}$ *is the largest subsequence of* α *that satisfies the above conditions.*

**Definition** **3.** *Suffix: The projection* $\alpha^{’}$ *of subsequence* $\beta =<e_{1}{}^{\prime }e_{2}{}^{\prime }\ldots e_{m}{}^{\prime }e_{m-1}{}^{\prime }>$ *for sequence* α *is* $\alpha^{\prime }=<e_{1}e_{2}\ldots e_{n}>$ *(n > m). The suffix of* β *for sequence* $\alpha^{’}$ *is* $<e_{m}{}^{\prime \prime }e_{m+1}\ldots e_{n}>$ *,* $e_{m}{}^{\prime \prime }=(e_{m}-e_{m}{}^{\prime })$ *.*

**Definition** **4.** *Projection database and projection database support: Let* α *be a sequence pattern in the sequence database S. The sequence* β *is prefixed with* α *. Then the projection database of* α *is the suffix of all* α *-prefixed sequences in S relative to* α *, denoted as* $S{|}_{\alpha }$ *. The support degree of* β *in* α *’s projection database* $S{|}_{\alpha }$ *meets the value of sequence* γ *with* β⊆α∗γ *.*

The PrefixSpan algorithm is a frequent pattern mining method that does not require candidates. The basic idea of this method is as follows: First find out each frequent item, then produce a collection of projection databases, each associated with a frequent item in the projection database. Next, excavate each database separately. The algorithm constructs a prefix pattern, which is connected to the suffix pattern to obtain frequent patterns, thereby avoiding the generation of candidates.

The following is an example of the sequence database *S* with min_sup = 2 to describe the mining process, as shown in Table 3.

(1) Obtain a sequence pattern of length 1. Scan S once to find all sequence patterns of length 1 in the sequence. They are <a>: 4, <b>: 4, <c>: 4, <d>: 3, <f>: 3. “<mode>: Count” indicates the mode and its support count.
(2) Divide the search space. The complete set of sequence patterns can be divided into the following six subsets based on six prefixes: The prefixes are subsets of <a>, <b>, <c>, <d>, <e> and <f> respectively.
(3) Find a subset of the sequence patterns. The subset of the sequence patterns mentioned in step 2 can be mined by constructing a corresponding projection database and mining each one recursively.

The sequence mode is shown in Table 4.

### 3.4. Bayesian Failure Network Model

The Bayesian network is a probabilistic graph model that represents the relationship between a series of random variables and variables in a directed acyclic graph. Faults in air handling units (AHUs) affect the building energy efficiency and indoor environmental quality significantly. There is still a lack of effective methods for diagnosing AHU faults automatically.

In Zhao’s 2017 study [21], a diagnostic Bayesian networks (DBNs)-based method was proposed to diagnose 28 faults, which cover most of the common faults in AHUs. Rear-end crash is one of the most common types of traffic crashes in the U.S. A good understanding of its characteristics and contributing factors is of practical importance. Previously, both multinomial logit models and Bayesian network methods have been used in crash modeling and analysis, respectively, although each of them has its own application restrictions and limitations. In Chen’s 2015 [22] study, a hybrid approach was developed to combine multinomial logit models and Bayesian network methods to comprehensively analyze driver injury severities in rear-end crashes based on state-wide crash data collected in New Mexico from 2010 to 2011.

In order to increase the diagnostic accuracy of the ground-source heat pump (GSHP) system, especially for multiple-simultaneous faults, Cai et al. [23] proposed a multi-source information fusion based fault diagnosis methodology by using Bayesian network, due to the fact that it is considered to be one of the most useful models in the field of probabilistic knowledge representation and reasoning, and can deal with the uncertainty problem of fault diagnosis well. The nodes of the graph represent random variables, and the directed edges from one (parent) node to another (child) node represent the relationship between the two node variables. The probability relationship between child nodes and parent nodes is represented by a conditional probability table.

The basic idea of the Bayesian network is to use probabilistic methods to deal with uncertainty in real life. It has a strong probabilistic reasoning ability and can learn rules from a large number of seemingly random and irregular data. After determining the structure and parameters of the Bayesian network, the Bayesian network model can be used to predict failure at specific input conditions.

One of the most important features of Bayesian networks is their ability to provide a good mathematical model for modeling complex relationships between random variables while maintaining a relatively simple visual presentation. They can be used to describe causal relationships between variables on a strict mathematical basis.

**Figure 4:**
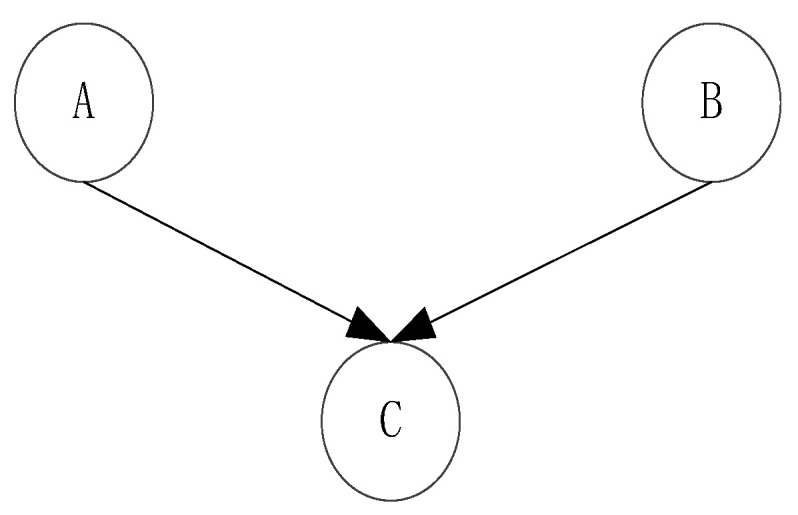
Head-to-head structure.

As shown in Figure 4. In the unknown case of C, A and B are independent, and this structure is called head-to-head condition independence. However C also depends on two random variables, A and B. The relationship between them can be expressed as:

(11) P(A,B,C)=P(C|A,B)P(A)P(B)

(12) P(A,B)=P(A)P(B)

**Figure 5:**
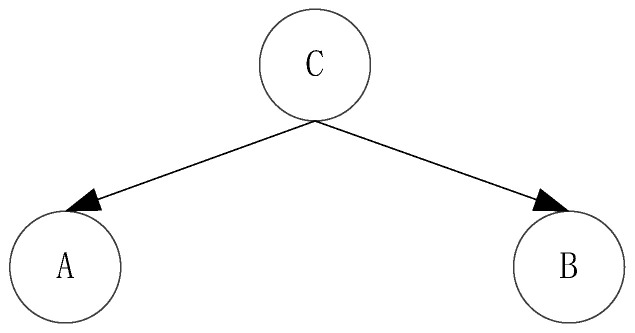
Tail-to-tail structure.

As shown in Figure 5. In the case of C which is given, A and B are independent. This structure is called a tail-to-tail condition independent structure. Both random variables A and B are dependent on C, so the relationship between them can be expressed as:

(13) P(A,B,C)=P(C)P(A|C)P(B|C)

(14) P(A,B,C)=P(A|C)P(B|C)

**Figure 6:**
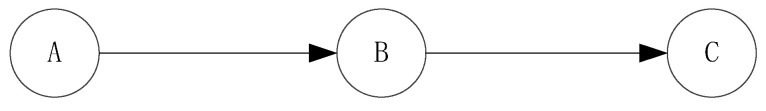
Head-to-tail structure.

As shown in Figure 6. In the case of B, which is given, A and C are independent. This structure is called head-to-tail condition independent; the head-to-tail structure can also be called a chained network. The variable B now depends on the variable A, while the random variable C depends on the variable B. The relationship between them can be expressed as:

(15) P(A,B,C)=P(A)P(B|A)P(C|B)

(16) P(A,C|B)=P(A|B)P(C|B)

Any complex Bayesian network can be formed by combining the three most basic forms of the network. The establishment of a Bayesian network is divided into two processes: structural learning and parameter learning. In the structural learning phase, the topological relationship between variables is determined by the sequence pattern. This is achieved by constructing a corresponding directed acyclic graph. The parameter learning phase involves the construction of a conditional probability table. If the value of each variable is directly observable, then the parameters of the network can be obtained directly. When the observations are complete, we use maximum likelihood estimation to obtain the parameters. Its log-likelihood function is:

(17) $$L=\frac{1}{N}\sum_{i=1}^{n}\sum_{i=1}^{s}\log(P(X_{i}|pa(X_{i}),D_{i}))$$

where $pa(X_{i})$ represents the dependent variable of $X_{i}$. $D_{i}$ represents the observed value. N represents total number of observations.

## 4. Results and Discussion

In this paper, 12,169 failure texts of four aircraft types were used as corpora for word vector training. According to the data, a total of 31 airplanes were selected, and 3727 failure texts were recorded for analysis. *R_ij_* (*i*, *j* = 1, 2, 3… 3727) indicates the distance between different texts. *S* denotes the similarity which is calculated by the word2vec moving distance model between faulty texts. The higher the similarity between the fault texts, the smaller the distance. So *R_ij_* = 1/*S_ij_* (*i*, *j* = 1, 2, 3, …, 3727). The same text distance is 0 and the distance is positive, as shown in Table 5.

**Figure 7:**
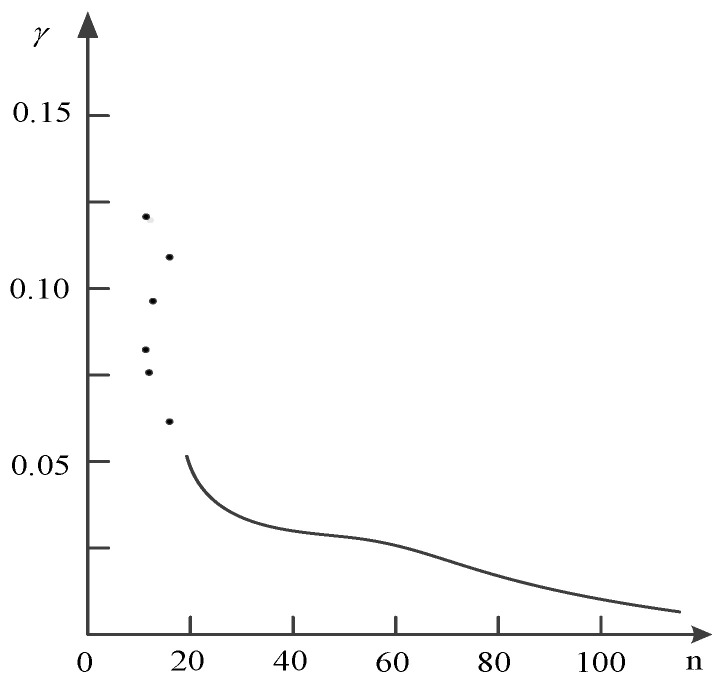
γ value.

According the above distance between different texts, Clustering by Fast Search and Find of Density Peaks (CFSFDP) is applied to clustering. γ is shown in Figure 7, which determines the six cluster centers.

There are six cluster centers: the 1062th, 1108th, 1743th, 3128th, 3145th, and 3693th. These six failures are as follows:

1062th: Transmitter failure;
1108th: The station received no signal;
1743th: The ground speed indicator is extremely small (0021) and does not move;
3128th: One starter generator starts overloaded and the signal light is on;
3145th: Engine internal grease;
3693th: “Land” position cannot be achieved due to high pressure.

After clustering, it is mainly divided into the above six types of faults. The six faults are very different from each other. The first failure is the transmitter failure. The second failure is the signal failure. This may include a variety of monitor signal failures. The third failure is the flight parameter indicator failure. The fourth failure is the generator failure, which is basically caused by generator overload and signal failure. The fifth failure is the engine internal failure. The sixth failure occurs when that other parts cannot accept high pressure. This may be due to fatigue.

Table 6 shows the clustering results:

Failure sequence mining was performed based on the above clustering results. The above six kinds of faults do not seem to have a clear logical relationship. However, the occurrence of one type of failure may cause another type of failure. To facilitate sequence mining, this paper uses words to indicate the above failures, as shown in Table 7.

According to the sequence mining algorithm, the sequence results are shown in Table 8.

**Figure 8:**
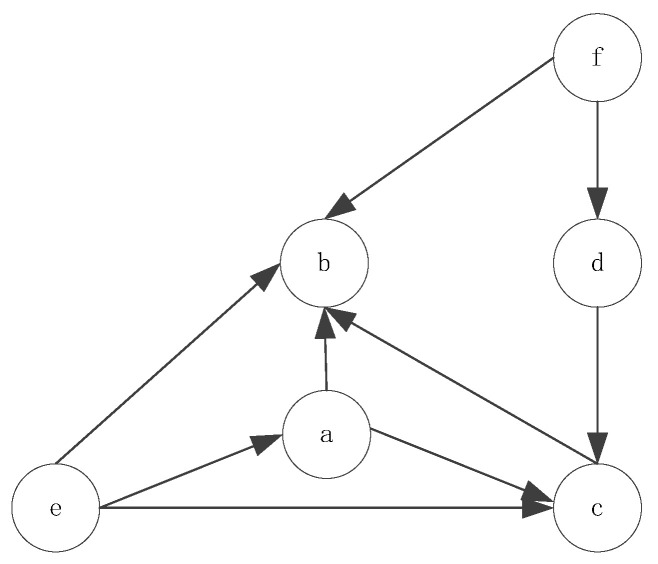
Bayesian network topology.

According to the above sequence results, the Bayesian network topology is shown in Figure 8.

Based on the above information, probability tables (Table 9, Table 10 and Table 11) are obtained. T represents occurrence, while F represents no occurrence.

In order to verify the accuracy of the forecasts, the first 1683 sequence records were extracted. As shown in Table 12, sequence algorithms were used to perform sequence mining, and the frequency of occurrence of each sequence was counted at the same time. Subsequently, the conditional probability table was used to make predictions. These two results were compared and the accuracy of the prediction was tested.

For these two sets of data, a fitting test was performed. The value of the goodness of fit was 0.921219. This value is very close to 1; the prediction accuracy is high.

## 5. Conclusions

In this paper, natural language processing knowledge was employed for data processing. Subsequently, the word2vec method was used for text vectorization. The clustering algorithm divided the failure types into six categories. A certain sequence relationship was found between the palms. The PrefixSpan algorithm was used to mine the sequence relationship. For failure prediction, the sequence is vital. According the above sequence relationship, a Bayesian failure network was successfully built based on textual failure data.

From Table 9, Table 10 and Table 11, under specified conditions, the Bayesian failure network was demonstrated to be able to predict the probability of the next type of failure to occur. For example, if engine failure, transmitter failure, and ground speed indicator failure occurred, wrong ‘Land’ position would subsequently occur. The occurrence probability of b was 0.62752. In the traditional method, failure prediction is based on structural data. However, unstructured data in a time series contains a lot of valuable information. The Bayesian failure network based on unstructured data can provide decision support for preventive maintenance.

This paper still has some deficiencies. For example, the proposed method roughly classified the above textual failures into six categories. The main inscription describes the network relationship between these six types of faults. For fault prediction, this represents a rough prediction. Subsequent research can refine the classification and increase the breakdown of fault classification. Furthermore, itcan provide guidance for the study of the cause of fault according to each mechanism. For future failure prediction work, a combination of structured data and unstructured data should be investigated in order to further improve the prediction accuracy.

## Figures and Tables

**Table 1:** Chinese word segmentation system comparison.

Segmentation System	User-Defined Dictionary	POS Tagging	Keywords Extraction	Support Traditional Chinese	Support UTF-8	New Word Recognition
jieba	√	√	√	√	√	√
Chinese Academy of Sciences	√	√	×	√	√	×
smallseg	√	×	×	√	×	√
snailseg	×	×	×	√	×	×

‘POS’ means ‘Part Of Speech’. ‘UTF’ means ‘Unicode Transformation Format’.

**Table 2:** Data preprocessing results.

Failure Text
motor/gasket/clutch/failure
switch/cargo hold/gate/chrome plating/aluminum layer/phase grinding/seepage/piston rod/deviation/center
temperature/seepage/unknown
radio station/sound/line/short circuit

**Table 3:** Sequence database.

Sequence ID	Sequence
1	<a(abc)(ac)d(cf)>
2	<(ad)c(bc)(ae)>
3	<(ef)(ab)(df)cb>
4	<e(af)cbc>

**Table 4:** Sequence mode.

Prefix	Projection Database	Sequence mode
<a>	<(abc)(ac)d(cf)> <(_d)c(bc)(ae)> <(_b)(df)eb> <(_f)cbc>	<a> <aa> <ab> <a(bc)> <a(bc)a> <aba> <abc> <(ab)> <(ab)c> <(ab)d> <(ab)f> <(ab)dc> <ac> <aca> <acb> <acc> <ad> <adc> <af>
<b>	<(_c)(ac)d(cf)> <(_c)(ae)> <(df)cb> <c>	<b> <ba> <bc> <(bc)> <(bc)a> <bd> <bdc> <bf>
<c>	<(ac)d(cf)> <(bc)(ae)> <b> <bc>	<c> <ca> <cb> <cc>
<d>	<(cf)> <c(bc)ae>	<d> <db> <dc> <dcb>
<e>	<(_f)cb> <(_f)(ab)(df)cb> <(af)cbc>	<e> <ea> <eab> <eac> <eacb> <eb> <ebc> <ec> <ecb> <ef> <efb> <efc> <efcb>
<f>	<(ab)(df)cb> <cbc>	<f> <fb> <fbc> <fc> <fcb>

‘Projection Database’ denotes the sequence which includes the prefix in the database. ‘_’ represents the prefix. ‘Sequence mode indicates the sequence meets the criteria.

**Table 5:** Distance.

Number	Distance	Number	Distance
*R*_11_	0.000	*R*_31_	2.044
*R*_12_	16.713	*R*_32_	1.897
*R*_13_	2.044	*R*_33_	0.000
*R*_14_	1.720	*R*_34_	2.003
*R*_15_	1.449	*R*_35_	1.843
*R*_16_	4.003	*R*_36_	12.083
*R*_21_	1.616	*R*_37_	1.973
*R*_22_	0.000	*R*_38_	17.443
*R*_23_	2.660	*R*_39_	3.243
*R*_24_	2.188	*R*_310_	10.657
*R*_25_	8.403	*R*_41_	1.720

**Table 6:** Statistics.

Number	Cluster Center	Total
1	1062	95
2	1108	1464
3	1743	60
4	3128	31
5	3145	35
6	3693	2042

‘Total’ indicates the number of failure text instances corresponding to the specific cluster.

**Table 7:** Representation.

Raw Data	1062	1108	1743	3128	3145	3693
Representation	a	b	c	d	e	f

a: Transmitter failure. b: Signal failure. c: Ground speed indicator failure. d: Generator failure. e: Engine failure. f: Wrong ‘Land’ position.

**Table 8:** Sequence results.

Number	Failure		Frequency
1	d	c	16
2	f	d	8
3	f	b	26
4	a	c	25
5	a	b	10
6	c	b	10
7	e	a	15
8	e	c	4
9	e	b	12

**Table 9:** Probability of related parameters.

Item	Probability
P(e)	0.00939
P(f)	0.54789
P(a = T|e = T)	0.42857
P(a = T|e = F)	0.02146
P(d = T|f = T)	0.00392
P(d = T|f = F)	0.00617

**Table 10:** Probability of c.

Item			Probability
d	a	e	P(c|d,a,e)
T	T	T	0.89357
F	T	T	0.37744
F	F	T	0.11429
F	T	F	0.26316
T	F	T	0.63041
T	F	F	0.51613
T	T	F	0.77929
F	F	F	0.00107

**Table 11:** Probability of b.

Item				Probability
e	a	c	f	P(b|e,a,c,f)
T	T	T	T	0.62752
F	T	T	T	0.28466
T	F	T	T	0.52226
F	F	T	T	0.17940
T	T	F	T	0.46085
F	T	F	T	0.11800
T	F	F	T	0.35559
F	F	F	T	0.01273
T	T	T	F	0.61479
F	T	T	F	0.27193
T	F	T	F	0.50952
T	F	F	F	0.34286
T	T	F	F	0.44812
F	T	F	F	0.10526
F	F	T	F	0.16667
F	F	F	F	0.37725

**Table 12:** Comparison of results.

Item	Actual Value	Predictive Value
a = T|e = T	8	6
a = T|e = F	27	28
d = T|f = T	4	3
d = T|f = F	16	10
c|d = T, a = T, e = T	22	21
c|d = F, a = T, e = T	14	8
c|d = F, a = F, e = T	2	1
c|d = F, a = T, e = F	12	2
c|d = T, a = F, e = T	10	11
c|d = T, a = F, e = F	8	2
c|d = T, a = T, e = F	20	7
c|d = F, a = F, e = F	4	2
b|e = T, a = T, c = T, f = T	29	33
b|e = F, a = T, c = T, f = T	23	26
b|e = T, a = F, c = T, f = T	11	7
b|e = F, a = F, c = T, f = T	18	16
b|e = T, a = T, c = F, f = T	24	22
b|e = F, a = T, c = F, f = T	18	16
b|e = T, a = F, c = F, f = T	19	12
b|e = F, a = F, c = F, f = T	13	16
b|e = T, a = T, c = T, f = F	16	18
b|e = F, a = T, c = T, f = F	10	9
b|e = T, a = F, c = T, f = F	11	12
b|e = T, a = F, c = F, f = F	6	9
b|e = T, a = T, c = F, f = F	11	16
b|e = F, a = T, c = F, f = F	5	5
b|e = F, a = F, c = T, f = F	5	6
b|e = F, a = F, c = F, f = F	582	622
